# Bioactive Power of Black Chokeberry Pomace as Affected by Advanced Extraction Techniques and Cryogrinding

**DOI:** 10.3390/molecules30163383

**Published:** 2025-08-14

**Authors:** Maja Repajić, Marija Zorić, Ivan Magnabosca, Sandra Pedisić, Verica Dragović-Uzelac, Ivona Elez Garofulić

**Affiliations:** University of Zagreb Faculty of Food Technology and Biotechnology, Pierottijeva 6, 10000 Zagreb, Croatia; maja.repajic@pbf.unizg.hr (M.R.); sandra.pedisic@pbf.unizg.hr (S.P.); verica.dragovic-uzelac@pbf.unizg.hr (V.D.-U.)

**Keywords:** *Aronia melanocarpa* L., valorization, PLE, MAE, UAE, optimization, polyphenols, anthocyanins, antioxidant, cryomilling

## Abstract

Black chokeberry (*Aronia melanocarpa* L.) pomace (BCP), a major by-product of juice production, is an underutilized source of polyphenols and anthocyanins with strong antioxidant properties. This study aimed to optimize and compare three green extraction techniques—pressurized liquid extraction (PLE), microwave-assisted extraction (MAE), and ultrasound-assisted extraction (UAE)—for recovering total polyphenols (TP) and total monomeric anthocyanins (TMA) from BCP, with reflux extraction as a benchmark. The effects of temperature, extraction time, and solid–solvent ratio were evaluated, and cryogrinding was assessed as a pre-treatment. PLE achieved the highest TP yields at elevated temperatures but reduced anthocyanin recovery, while MAE offered a balance of high TP and TMA, with strong antioxidant capacity. Cryogrinding enhanced TP extraction, with only 1 min of cryogrinding maximizing yield. UPLC-MS/MS analysis of optimized MAE extract confirmed cyanidin-3-glucoside and cyanidin-3-galactoside as dominant anthocyanins, alongside notable flavonols and phenolic acids, validating the rich phenolic profile. Overall, MAE combined with 1 min of cryogrinding proved to be the most effective approach for preserving heat-sensitive compounds while achieving high yields. These findings demonstrate that optimized green extraction can efficiently valorize BCP, supporting sustainable food processing and waste reduction in line with circular economy principles.

## 1. Introduction

The increasing consumer demand for natural antioxidants and functional ingredients has intensified interest in the sustainable sourcing of bioactive compounds from food industry by-products. Among these, black chokeberry (*Aronia melanocarpa* L.) stands out as a unique raw material due to its exceptionally high content of polyphenolic compounds, particularly anthocyanins, proanthocyanidins, phenolic acids, and flavonols. These compounds are well documented for their strong antioxidant activity, radical scavenging properties, and associated health benefits, including anti-inflammatory, cardioprotective, and anti-carcinogenic effects [1].

Black chokeberry is widely processed into juices, syrups, and concentrates, generating substantial amounts of pomace as a by-product. This pomace consists predominantly of berry skins, seeds, and residual pulp and retains a considerable proportion of the fruit’s original phenolic content. According to Kaloudi et al. [2], only 13% of the fruit’s total phenolic content is transferred to the juice during pressing, 10% of the total anthocyanins, 66% of the phenolic acids, and 23% of the flavonoids, indicating that a considerable amount, comprising predominantly anthocyanins and flavonoids, remains in the pomace and could be further extracted for valorization. Indeed, previous studies have characterized black chokeberry pomace (BCP) as the main source of anthocyanins such as cyanidin-3-galactoside and cyanidin-3-glucoside, flavonols (e.g., quercetin and kaempferol glycosides), and phenolic acids (e.g., chlorogenic and neochlorogenic acids), which contribute synergistically to its antioxidant potential [2,3,4,5].

Despite its high value, BCP remains largely underutilized and is often discarded or used for low-value applications such as animal feed or compost. In the context of sustainable production and circular economy principles, the valorization of this bio-residue into valuable antioxidant extracts represents a promising strategy to reduce food waste and add economic value to processing chains. However, the effective recovery of these sensitive compounds depends greatly on the choice of extraction method and the optimization of critical process parameters.

Traditional extraction approaches, including reflux or maceration, typically require prolonged extraction times, elevated temperatures, and high solvent consumption, which can lead to the degradation of heat-sensitive anthocyanins and increase energy costs [6]. In contrast, green extraction technologies such as pressurized liquid extraction (PLE), microwave-assisted extraction (MAE), and ultrasound-assisted extraction (UAE) offer significant advantages by enhancing extraction efficiency, reducing process time and solvent usage, and minimizing thermal degradation of target compounds [7]. The efficiency of these advanced methods, however, is highly dependent on the precise control of key operational parameters. For instance, temperature has been shown to play a dual role: elevated temperatures can improve the solubility and mass transfer of phenolics, but may simultaneously degrade thermolabile anthocyanins, as demonstrated in studies on grape and blackberry pomace [8,9]. Similarly, extraction time must be carefully optimized to balance sufficient cell wall disruption with minimal compound degradation. The solid–solvent ratio (SSR) is another critical factor; increasing the SSR generally enhances extraction efficiency by providing more solvent per unit of sample, thus improving diffusion rates and compound solubilization until extraction equilibrium is reached [10].

Furthermore, mechanical pre-treatments such as cryogrinding have gained attention as simple yet effective methods to increase extraction yields by reducing particle size, which increases the available surface area for mass transfer and facilitates solvent penetration into plant matrices [11]. Recent studies have shown that cryogrinding can significantly reduce particle size and enhance phenolic recovery from myrtle leaves [12], fenugreek seeds [13], and sword beans [14].

Although the separate application of green extraction techniques and mechanical pre-treatments has been widely explored, comprehensive studies comparing the combined effects of optimized PLE, MAE, and UAE parameters, together with cryogrinding, for maximizing both total polyphenols and anthocyanins from BCP remain scarce.

Therefore, the aim of this study was to systematically investigate the influence of critical extraction parameters, temperature, extraction time, and SSR on the recovery of total polyphenols and total monomeric anthocyanins from BCP using PLE, MAE, and UAE. Furthermore, the study assessed the additional impact of cryogrinding as a pre-treatment to maximize extraction efficiency. The resulting extract was characterized for individual phenolic composition, and the antioxidant capacity was evaluated through multiple assays to provide a comprehensive understanding of its functional potential. By optimizing and comparing these techniques, this work seeks to provide a practical basis for the sustainable valorization of BCP, demonstrating its potential as a high-value source of natural antioxidants and contributing to the broader goals of food waste reduction and a circular bioeconomy.

## 2. Results and Discussion

### 2.1. Determination of PLE, MAE, and UAE Optimal Process Conditions

The results of total polyphenol (TP) and total monomeric anthocyanin (TMA) content determination in BCP extracts obtained under different process conditions of PLE, MAE, and UAE are given in Table 1.

The TP content ranged from 86.0 to 132.5 mg gallic acid equivalents (GAE)/g of dry matter (dm) under PLE conditions, from 58.5 to 95.7 mg GAE/g dm under MAE conditions, and from 63.1 to 88.9 mg GAE/g dm under UAE conditions. Woźniak et al. [15] reported a TP content ranging from 3.67 to 15.21 mg GAE/g of thawed chokeberry pomace, using supercritical carbon dioxide extraction with ethanol under varying conditions of temperature, pressure, and ethanol concentration. In comparison, the TP values obtained in this study using PLE were 88–96% higher, indicating that PLE is a more efficient method for extracting phenolic compounds from BCP than supercritical carbon dioxide extraction. Simić et al. [16] reported TP contents ranging from 3.73 to 4.58 mg GAE/g of frozen chokeberries when studying the impact of MAE exposure time from 5 to 15 min and microwave power ranging from 300–600 W on TP content in chokeberry fruit, which is significantly lower (94–95%) than the TP content found in this study. However, the mentioned study employed an extraction approach with no temperature control, so excessive degradation of heat sensitive compounds could have occurred. It should also be noted that none of the above studies used dried chokeberry material. As for UAE, slightly comparable values (16.85 to 74.28 mg GAE/g dm, i.e., 16–73% lower) to those obtained in this work were reported by D’Alessandro et al. [17], who investigated the TP content in dried BCP extracts obtained by UAE while evaluating the influence of extraction time (0–240 min), temperature (20–70 °C), solvent composition (0–50% ethanol in water), and ultrasound power (0–100 W).

Observed ranges for TMA content were 8.2–24.1 mg cyanidin-3-glucoside equivalents (C3GE)/g dm in PLE extracts, 20.3–26.6 mg C3GE/g dm in MAE extracts, and 18.7–23.2 mg C3GE/g dm in UAE extracts. Roda-Serrat et al. [18] reported anthocyanin contents of 18.1–51.0 mg/dry weight in chokeberry pomace using homogenization in citric acid aqueous solution (0.25, 0.75, and 1.5%, *w*/*v*), which is 53–55% higher compared to the PLE yields obtained in this study. Elez Garofulić et al. [3] reported 79–83% lower anthocyanin contents (3.35–5.53 mg/g) in thawed BCP pomace using MAE under various irradiation time (4, 6, 8, and 10 min) and temperature (40, 60, and 80 °C) combinations. They also applied UAE under different amplitudes (25, 50, and 75%) and sonication times (4, 6, 8, and 10 min), achieving TMA yields of 3.89–5.67 mg/g, which is 75–79% lower than the UAE yields obtained in this study. In addition to the use of undried material, their high SSR (1:100 g/mL) may have limited extraction efficiency compared to the values obtained under MAE and UAE in this study.

The influence of individual PLE, MAE, and UAE process parameters on TP and TMA in BCP extracts is shown in Table 2. The presented results indicate that temperature had a statistically significant effect on TP and TMA content during PLE, while there was no observed effect of static extraction time and SSR. The highest average value of TP content was achieved in the extract obtained at 150 °C, while there was no statistically significant difference between the TP content extracted at 100 and 125 °C. This suggests that the successful extraction of phenolic compounds from BCP favors the use of higher temperatures. Pereira et al. [8] conducted PLE on grape pomace at temperatures of 40, 60, 80, and 100 °C using different solvents and an extraction time of 4 h. Using a 50% aqueous ethanol solution as the extraction solvent, they observed that increasing the temperature resulted in a significant increase in TP content. Similarly, Dobroslavić et al. [19] observed an increase in TP content with increasing temperature. They performed PLE on bay leaves at 90, 120, and 150 °C and concluded that the TP content increased proportionally with the rise in temperature. However, in the extraction of anthocyanins, the opposite trend can be observed. The highest average content of TMA was recorded in the extract obtained at 100 °C, and with each further increase in temperature, their content decreased, with the lowest value observed in the extract obtained at 150 °C. According to the previously mentioned study by Pereira et al. [8], increasing the PLE temperature from 40 to 100 °C also resulted in a decrease in the TMA content in grape pomace extracts.

This trend suggests that raising the temperature above 100 °C negatively affects the yield of anthocyanins, which is understandable considering that anthocyanins are thermolabile and degrade when exposed to elevated temperatures. Similar results were reported by Ju and Howard [20] in their study, in which they conducted PLE on dried grape skin at temperatures ranging from 20 to 140 °C using various solvents. When a 60% aqueous ethanol solution containing 0.1% hydrochloric acid was used as an extraction solvent, the TMA content decreased with increasing temperature, while the TP content increased. Da Fonseca Machado et al. [9] performed PLE on blackberry pomace using different solvents at 60, 80, and 100 °C. In their study, they also observed that higher temperatures led to an increase in TP content, while the TMA content decreased. The difference between the loss of anthocyanins at elevated extraction temperatures and the increased yield of TP was attributed to the enhanced extraction of more thermostable compounds, such as procyanidins and phenolic acids.

Considering the obtained results and observed effects, it was concluded that optimal PLE process conditions for the simultaneous extraction of both TP and TMA were temperature 125 °C, static time 5 min, and SSR 1:40 g/mL.

During the MAE, temperature and extraction time significantly affected the TP content of BCP extracts, with no observed effect on anthocyanins, while the SSR significantly affected both TP and TMA content. The highest average value of TP content was achieved at 80 °C. This value was statistically significantly higher than that obtained at 40 °C, but did not differ significantly from the value obtained at 60 °C, indicating that further temperature increases above 60 °C did not have a significant effect on TP content. Wu et al. [21] investigated the use of MAE for isolating phenolic compounds from potatoes at temperatures of 50, 60, 70, and 80 °C. They observed a significant increase in TP concentration with rising temperature. A similar pattern was reported by Jokić et al. [22] during the optimization of MAE for phenolic extraction from broccoli. In their study, the concentration of extracted phenolics increased as the temperature rose from 50 to 73 °C, while further heating to 90 °C resulted in a decrease in TP content. Zhao et al. [23] also observed a comparable trend when applying MAE to *Melastoma sanguineum*. Increasing the temperature from 20 to 50 °C led to a significant rise in TP content, whereas a further increase to 70 °C did not produce a statistically significant change.

Furthermore, extending the irradiation time from 5 to 10 min resulted in an increase in TP content. As previously mentioned, Zhao et al. [23] also examined the effect of MAE irradiation time on TP yield. Although their extraction times were considerably longer than those used in this study, they similarly observed that extending the irradiation time from 15 to 45 min positively influenced TP yield. However, further prolongation to 60 min led to a slight decrease in TP content.

The SSR had a statistically significant effect on all observed dependent variables. The highest average values for TP and TMA content were achieved at an SSR of 1:80 g/mL. The lowest values for both variables were recorded at a ratio of 1:40 g/mL. However, statistical analysis revealed no significant difference in TP content between the 1:60 and 1:80 g/mL ratios, suggesting that a 1:60 g/mL ratio is sufficient for effective extraction of TP from BCP using MAE. In contrast, for maximum TMA yield, the highest SSR (1:80 g/mL) should be considered. A similar trend was observed in the study by Wen et al. [24], who applied MAE to blackberry fruits. They reported that increasing the SSR (1:15, 1:20, and 1:25 g/mL) led to an increase in TP content. Pap et al. [25] also found that increasing the SSR (from 1:10 to 1:20 g/mL) positively influenced anthocyanin extraction from black currants using MAE. Likewise, Zhao et al. [23], in their MAE study on *M. sanguineum*, recorded a significant increase in TP content with increasing SSR (1:10, 1:20, and 1:30 g/mL). However, further increases to 1:40, 1:50, and 1:60 g/mL led to a slight decrease in phenolic yield, suggesting that extraction equilibrium in mass transfer was likely reached at a ratio of 1:30 g/mL.

Considering the statistical analysis of all examined dependent variables, the optimal conditions for efficient MAE of TP and TMA from BCP were determined to be a temperature of 80 °C, an extraction time of 10 min, and an SSR of 1:80 g/mL.

When applying UAE as an extraction method, amplitude did not exhibit a statistically significant effect on any of the observed dependent variables, with values remaining consistent across all amplitudes tested. In line with the findings of Elez Garofulić et al. [3], who also investigated UAE from BCP, increasing the amplitude from 25 to 75% did not result in a statistically significant change in the yield of TMA. Similarly, Borrás-Enríquez et al. [26], who applied UAE to mango by-products, reported no significant differences in TP content across amplitudes ranging from 30 to 90%.

Extraction time demonstrated a statistically significant effect only on the content of TMA, with a shorter extraction duration (5 min), proving more effective for anthocyanin recovery. Comparable findings were reported by González et al. [27], who examined the impact of extraction time (2–25 min) on anthocyanin yield from blackcurrant using UAE. Their results also indicated that a shorter extraction time (5 min) yielded higher anthocyanin content, whereas extended durations (10–20 min) led to reduced anthocyanin yields, likely due to degradation.

The SSR had a statistically significant influence on TP content, with no effect on TMA. The highest average TP content was achieved at SSRs of 1:60 and 1:80 g/mL, while the lowest was recorded at 1:40 g/mL. These results indicate that increasing solvent volume enhances phenolic extraction efficiency. However, further increases beyond a 1:60 g/mL ratio did not result in statistically significant improvements in phenolic yield. A similar trend was observed by He et al. [28] during UAE of blueberry pomace, where increasing the SSR from 1:15 to 1:20 g/mL improved TP content, but a ratio of 1:25 g/mL did not yield a significant additional benefit. D’Alessandro et al. [17] reported comparable results for UAE applied to BCP, where increasing the SSR from 1:10 to 1:20 g/mL enhanced phenolic recovery, but a further increase to 1:40 g/mL did not lead to a significant improvement in yield.

Considering all the observations above, optimal UAE parameters for simultaneous recovery of TP and TMA were set at amplitude 50%, extraction time 5 min, and SSR 1:60 g/mL.

**Figure 1:**
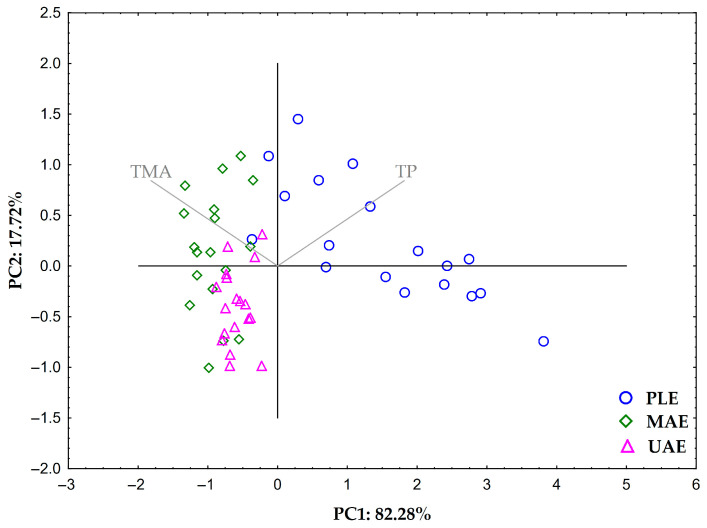
Distribution of BCP extracts in a two-dimensional coordinate system defined by the first two principal components (PC1 and PC2) in relation to the type of extraction technique (BCP—black chokeberry pomace; PLE—pressurized liquid extraction; MAE—microwave-assisted extraction; UAE—ultrasound-assisted extraction; TP—total polyphenols; TMA—total monomeric anthocyanins).

Finally, to examine the possible grouping of the obtained BCP extracts according to the content of TP and TMA with respect to the applied extraction technique, principal component analysis (PCA) was performed. The results are shown in Figure 1.

The communality values of TP and TMA exceeded 0.8, indicating that both variables are well represented by the extracted components and contribute significantly to the factor structure. The distribution of the samples was examined in the space defined by the first two principal components (PC1 and PC2), both of which had eigenvalues greater than 1. Together, PC1 and PC2 explained 100.00% of the total variance in the data. A very strong correlation was observed between PC1 and TP (r > 0.9), as well as between PC1 and TMA (r < − 0.9). In contrast, moderate correlations (r > 0.4) were found between PC2 and both TP and TMA. The results reveal a clear grouping of extract samples according to the extraction technique. Almost all samples obtained using PLE were located in the area of positive PC1 values and were characterized by high TP content. In contrast, all samples obtained using MAE and UAE clustered in the area of negative PC1 values, characterized by high levels of TMA. These results confirm earlier observations showing clear differences in the efficiency of PLE, MAE, and UAE in isolating phenolic compounds and anthocyanins from BCP, with PLE demonstrating the highest efficiency in the isolation of phenolic compounds. However, the high temperature applied during PLE (≥ 100 °C) is unfavorable for anthocyanin isolation due to its thermolability, which justifies the use of MAE. The milder conditions applied during MAE, particularly the temperature (max. 80 °C), preserved anthocyanins while still achieving a satisfactory yield of phenolic compounds. Therefore, MAE can be highlighted as an effective method for obtaining BCP extracts rich in both phenolic compounds and anthocyanins.

### 2.2. Efficiency Comparison of PLE, MAE, UAE, and Reflux

Optimized extraction techniques (PLE, MAE, and UAE) were compared with reflux as a conventional extraction method in terms of TP and TMA yield and antioxidant capacity of obtained extracts determined by multiple assays (FRAP, DPPH, ABTS, and ORAC), each capturing different mechanisms of antioxidant activity (Table 3).

Reflux extraction yielded the highest TP content (120.8 mg GAE/g dm), followed by PLE and MAE, with 101.3 and 94.8 mg GAE/g dm, respectively, indicating a limitation of ultrasound-based methods for phenolic recovery under the tested conditions. These results are consistent with previous reports where thermal methods enhanced phenolic solubility and matrix softening, leading to increased extraction yields [29].

However, the highest TMA content was recorded in MAE extracts (24.0 mg C3GE/g dm), followed closely by UAE and reflux, while PLE yielded significantly less (19.3 mg C3GE/g dm). This suggests that MAE may be particularly effective for extracting thermolabile anthocyanins due to shorter processing times and lower temperatures, unlike more intensive processes such as PLE. This also aligns with previous studies indicating that microwave heating favors the extraction of anthocyanins due to rapid cell wall rupture and reduced exposure to elevated temperatures [6].

Regarding antioxidant capacity, different trends were observed in relation to the applied assay. The FRAP assay, which reflects electron-donating ability, showed that PLE (1052.1 µmol TE/g dm) and reflux (1033.4 µmol TE/g dm) extractions were superior, with no significant difference between them. These results correlate with their high TP content, underscoring the strong reducing capacity of polyphenol-rich extracts. MAE (983.7 µmol TE/g dm) also showed comparable FRAP values, while UAE was significantly lower (637.4 µmol TE/g dm), aligning with its reduced TP content. In the DPPH assay, which is also based on single electron transfer, MAE and reflux outperformed PLE and UAE, yielding values of 420.4 and 472.9 µmol TE/g dm, respectively. The ABTS assay revealed significantly higher values in MAE (681.3 µmol TE/g dm) and reflux (790.2 µmol TE/g dm) extracts. PLE and UAE showed moderate ABTS values (519.2 and 461.3 µmol TE/g dm, respectively). This trend in ABTS values may only be partially related to TMA content. The literature data supports the observation that anthocyanins, particularly cyanidin derivatives, are efficient ABTS radical scavengers due to their structural features, specifically hydroxylation and conjugation patterns that stabilize radical intermediates [30]. However, the results obtained indicate a possible interference of other non-phenolic compounds in the ABTS assay. Some of the possible interactions could be those of lipophilic compounds present in BCP that to a certain extent partition into alcohol-based solvents during the extraction with ABTS radical, as ABTS assay is known to detect both hydrophilic and some lipophilic antioxidants, unlike the FRAP, DPPH, and ORAC assay, when performed in the hydrophilic mode [31]. The ORAC results followed a trend similar to ABTS: reflux extraction yielded the highest ORAC value (222.6 µmol TE/g dm), followed closely by MAE (199.6 µmol TE/g dm), both significantly surpassing UAE (142.7 µmol TE/g dm) and especially PLE (92.2 µmol TE/g dm). These findings indicate that conventional and microwave-based methods may preserve peroxyl radical-scavenging capacity better than pressurized and ultrasound-assisted methods, possibly due to less structural degradation of antioxidant molecules. This observation can also be attributed to TMA content. Anthocyanins, particularly cyanidin derivatives, are well-known to contribute significantly to ORAC values because their ortho-dihydroxy B-ring structure and conjugated system make them good hydrogen donors. This structural feature makes them more reactive in HAT-based assays (like ORAC) than in some SET-based assays, such as FRAPP and DPPH [32]. Accordingly, Wu et al. [33] showed that black chokeberry’s high ORAC values are strongly associated with its high levels of cyanidin glycosides.

In summary, while PLE maximized TP yield and demonstrated strong electron transfer capacity in FRAP assay similar to conventional reflux extraction, MAE proved to be the most balanced advanced technique overall in relation to TP and TMA content and antioxidant capacity.

### 2.3. Effects of Cryogrinding

In order to evaluate cryogrinding as a pre-treatment for maximizing the extraction yield, samples of BCP were ground using a conventional method, marked as 0 min cryogrinding time, as well as by cryogrinding for different durations, namely 1, 3, 5, 7, and 9 min. The results of the particle size distribution measurement are shown in Table 4.

The highest value for the d(0.1) parameter was recorded in the sample ground using the conventional grinding method, while the lowest value was observed in the sample after 9 min of cryogrinding. It is evident that the d(0.1) values decrease with increasing grinding time, demonstrating the effectiveness of cryogrinding in reducing the size of 10% of the particles. Notably, just 1 min of cryogrinding was sufficient to reduce 10% of the particles by 86.5% compared to conventional grinding. However, further extension of grinding time did not result in statistically significant differences in d(0.1) values among the cryogenically ground samples. Similar findings were reported by Kraljić et al. [11], who performed cryogrinding of rapeseed press cake for 2, 4, 8, and 12 min. The lowest d(0.1) value was likewise recorded in the sample ground for the longest duration (12 min), resulting in a reduction of 10% of the particles by 89.23% compared to the conventionally ground sample. Regarding the values for d(0.5), the highest value was again found in the conventionally ground sample, while the lowest was observed in the sample after 9 min of cryogrinding. A statistically significant difference in the d(0.5) parameter was found between the conventionally ground sample and those cryogenically ground for 1, 3, and 5 min, with the 50% particle size being reduced by 68.4%, 77.7%, and even 89.5% after 1, 3, and 5 min of cryogrinding, respectively. Further extension of the grinding time to 7 or 9 min did not lead to a significant additional reduction in the 50% particle size. Kraljić et al. [11] also reported a decrease in d(0.5) values with prolonged cryogrinding, observing statistically significant differences among all samples compared to the conventional sample, with reductions in the 50% particle size of 56.28, 65.09, 76.55, and 84.30% after 2, 4, 8, and 12 min of cryogrinding, respectively. The 90% particle size (d(0.9)) followed the same trend as the previously discussed parameters: the highest value was recorded in the conventionally ground sample, and the lowest in the sample ground cryogenically for 9 min. Statistical analysis showed results similar to those for d(0.5), except that no significant difference was observed in the 90% particle size for the samples ground cryogenically for 1 and 3 min. The results indicate that the reduction in the 90% particle size was 36.0% and 41.1% after 1 and 3 min of cryogrinding compared to conventional grinding, while 5 min of cryogrinding reduced the 90% particle size by 65.6%. Further extension of cryogrinding to 7 and 9 min did not contribute to a statistically significant additional reduction in the 90% particle size. In contrast to the d(0.9) values obtained in this study, all d(0.9) values in the work by Kraljić et al. [11] showed statistically significant differences. They recorded the lowest value in the sample subjected to the longest cryogrinding (12 min), with a reduction of 90% of the particles by 83.12% compared to the conventionally ground sample.

The final parameter examined was the particle size distribution span, which describes the homogeneity of the particle size distribution and should be at the lowest values, thereby indicating good uniformity of the particle size. The calculated span values ranged from 2.8 to 9.7 µm, with the highest span recorded in the sample after 5 min of cryogenic grinding and the lowest in the conventionally ground sample. Given the higher span values in the cryogenically ground samples, it can be concluded that the particle distribution in these samples was less homogeneous, whereas the greatest uniformity was observed in the conventionally ground sample. Kraljić et al. [11] reported the lowest span value in the sample obtained after 4 min of cryogenic grinding, indicating the highest uniformity in that sample. Conversely, the lowest homogeneity was observed in the sample ground for the longest duration (12 min), which was also confirmed in this study.

Considering all the particle size parameters discussed, it can be concluded that cryogrinding is highly effective in significantly reducing the particle size of BCP. A maximum of 5 min of this pre-treatment, or even less, is sufficient to achieve a substantial particle size reduction and thereby increase the surface available for efficient mass and energy transfer during the extraction.

The effect of different cryogrinding exposure times on the content of TP and TMA in BCP extracts obtained under optimized MAE conditions (temperature 80 °C, extraction time 10 min, and SSR 1:80 g/mL) as well as on the antioxidant capacity of extracts is shown in Table 5.

The duration of cryogrinding had a statistically significant impact on TP content as well as the antioxidant capacity measured by FRAP, DPPH, ABTS, and ORAC assays, whereas no statistically significant differences were observed in the TMA content between samples with respect to cryogrinding time. This can be explained by the fact that most anthocyanins are located in the fruit skin [34], and thus, their majority was likely released even during conventional grinding of BCP. This is supported by the similar TMA contents obtained from the conventionally ground sample subjected to MAE under optimal conditions and the average values of all cryogenically ground samples (24.0 vs. 22.2 mg C-3-GE/g dm).

The highest TP content (115.1 mg GAE/g dm) was recorded in the sample cryogenically ground for 1 min, while the lowest content (99.1 mg GAE/g dm) was observed after 7 min of cryogenic grinding. Since the 1 min cryoground sample exhibited a statistically significant higher TP content compared to all other samples, this pre-treatment could be considered the most effective for phenolic compound isolation. Compared to the conventionally ground sample, the superiority of cryogrinding was shown through a 17.6% increase in TP content (115.1 vs. 94.8 mg GAE/g dm).

Contrasting results were reported by Balbino et al. [35], who investigated cryogrinding as a pre-treatment to enhance the extraction of bioactive molecules from pumpkin seed cake. Their cryogrinding durations were 4, 8, and 12 min, and they observed increasing phenolic yields with longer grinding times, with the highest yield at 12 min. These differences may arise from the distinct structural properties of the materials. Pumpkin seed cake possesses a tougher, fibrous matrix that requires longer grinding to disrupt effectively and release entrapped phenolics [35]. In contrast, the softer structure of BCP likely allows rapid release of phenolics, rendering longer grinding times less effective.

Significantly higher antioxidant capacity determined by FRAP, DPPH, and ABTS assays were observed in samples ground for longer than 5 min. Specifically, the highest FRAP values were measured in samples ground for 5 and 9 min, the highest DPPH values in samples ground for 9 min, and the highest ABTS values in samples ground for 7 and 9 min. Prolonged cryogrinding likely contributed to the release of additional bioactive compounds in BCP, such as ascorbic acid or proanthocyanidins (condensed tannins), enhancing antioxidant potential. Polymeric proanthocyanidins often exhibit higher antioxidant activity than monomeric forms in assays such as DPPH and FRAP [36]. However, their contribution to TP content, as measured by the Folin–Ciocalteu assay, can be underestimated due to both structural and methodological limitations. The FC reagent reacts with a wide range of reducing substances and phenols, but its response can vary depending on phenolic structure. As a result, proanthocyanidins may react less predictably or intensely, and if they are poorly extracted, their actual contribution to TP content may not be fully captured by the assay [37]. However, it is notable that antioxidant capacity values measured by all three assays were comparable between the conventionally ground sample and those ground cryogenically (FRAP: 983.6 vs. 945.2 µmol TE/g dm; DPPH: 420.4 vs. 411.7 µmol TE/g dm; ABTS: 681.3 vs. 718.1 µmol TE/g dm). Balbino et al. [35] reported increased antioxidant capacity (measured by DPPH assay) in pumpkin seed cake samples subjected to the longest cryogenic grinding time (12 min) compared to the control. Rosa et al. [38] studied wheat bran extracts and found that antioxidant capacity (measured by ABTS assay) significantly increased with cryogenic grinding time from 3 to 20 min, with the highest value after 20 min. These results align with the trends observed in the present study.

On the other hand, the antioxidant capacity measured by ORAC assay showed the opposite trend, namely a decrease in antioxidant capacity with prolongation of cryogrinding time. A possible explanation for the observed decrease in antioxidant capacity measured by the ORAC assay, in line with the trend for TP content, may lie in the nature of the assay itself. The ORAC method is highly sensitive to free, low-molecular-weight phenolic compounds that act predominantly through hydrogen atom transfer mechanisms [32]. Mechanical grinding of the samples and, by extension, prolonged cryogrinding, although effective for cell disruption, may promote partial oxidation or polymerization of phenolics due to mechanical stress [39], leading to a reduction in extractable free phenolics. Consequently, while electron transfer-based assays such as FRAP, DPPH, and ABTS can still detect antioxidant capacity in polymerized or oxidized phenolics, the ORAC assay reflects a decrease due to the loss of these labile antioxidants.

In conclusion, cryogrinding proved to be an effective pre-treatment for the isolation of phenolic compounds from BCP, with only 1 min of grinding required to achieve maximum phenolic yield.

### 2.4. Phenolic Profile of BCP Extract Obtained Under Optimal MAE Conditions in Combination with Cryogrinding

The extract of BCP obtained under the optimal MAE conditions (80 °C/10 min/1:80 g/mL) with a cryogrinding pre-treatment of 1 min was further analyzed for individual phenolic compound composition using the UPLC-MS/MS method, and the results are presented in Table 6.

In total, 21 phenolic compounds were identified, including 4 anthocyanins, 3 phenolic acids, 12 flavonols, 1 flavan-3-ol, and 1 procyanidin. Anthocyanins were the dominant group, accounting for 64.16 mg/g dm (66.9%). These findings align with Kaloudi et al. [2], who reported that anthocyanins constituted the largest proportion of TP in chokeberry skin (66%) compared to chokeberry flesh with seeds (10.8%) and whole dried berries (27%). They concluded that chokeberry skin contains up to 73% of the total fruit anthocyanins. Oszmiański and Lachowicz [4] also found that anthocyanins made up about 50% of the TP extracted from ground freeze-dried BCP, which is slightly lower than the proportion observed in this study. This difference may result from using different chokeberry cultivars or different pomace production as well as extraction methods.

Among the identified anthocyanins, cyanidin-3-glucoside and cyanidin-3-galactoside were the most abundant (20.81 and 20.67 mg/g dm, respectively), followed by cyanidin-3-arabinoside and cyanidin-3-xyloside, present in similar concentrations (11.37 and 11.31 mg/g dm). The distribution pattern only partly agrees with the results of Oszmiański and Lachowicz [4], who identified cyanidin-3-*O*-galactoside as the predominant anthocyanin in chokeberry pomace powder (7961.70 mg/100 g dm), followed by cyanidin-3-*O*-arabinoside (3116.02 mg/100 g dm). The same study also reported cyanidin-3-*O*-xyloside and cyanidin-3-*O*-glucoside at lower concentrations. Other studies have also confirmed cyanidin-3-*O*-arabinoside as the second most abundant anthocyanin in chokeberry products [2,40,41]. Conversely, the findings of the present study as well as the previous study of our research group [3] showed cyanidin-3-*O*-glucoside to be similar in concentration to cyanidin-3-*O*-galactoside, thereby being one of two major anthocyanins in BCP. The differences observed across studies may be attributed to cultivar variation and processing conditions. Notably, both this study and Elez Garofulić et al. [3] used the ‘Nero’ cultivar from the same source, explaining the similarity in anthocyanin profiles. However, Elez Garofulić et al. [3] reported a considerably lower total anthocyanin concentration (5.36 mg/g dm) than that determined in this study (64.16 mg/g dm). This discrepancy could be due to differences in pomace preparation; their pomace was produced using industrial pressing, which likely transferred a higher proportion of anthocyanins into the juice, leaving less in the pomace. Moreover, the pomace analyzed presently was freeze-dried, whereas a previous study used frozen pomace, which may further explain the higher concentrations obtained in this research.

When comparing the total anthocyanin content determined by spectrophotometric and UPLC-MS/MS analyses, a significant difference was noted; the UPLC-MS/MS method yielded three-fold higher anthocyanin levels. This can be explained by methodological differences. The pH differential method quantifies only monomeric anthocyanins by exploiting their reversible structural transformation under different pH conditions, while UPLC-MS/MS, being more sensitive and precise, detects and quantifies individual anthocyanin compounds, including minor or structurally complex derivatives that spectrophotometric methods typically fail to detect, and sums their concentrations. Similar findings were reported by Vagiri and Jensen [42], who observed that total anthocyanins in chokeberry pomace determined by chromatography were, on average, twice as high as TMA determined spectrophotometrically.

Flavonols represented the second-largest group (29.08 mg/g dm; 30.3% of identified phenolics). Kaempferol-3-glucoside was the most abundant (24.53 mg/g dm), with noticeable amounts of quercetin-3-glucoside and quercetin-3-galactoside, followed by quercetin-3-rutinoside, quercetin-3-vicianoside, and quercetin-3-dihexoside. Other flavonols were present at concentrations below 0.1 mg/g dm. Sójka et al. [5] reported flavonols as the least abundant group (0.86%) in chokeberry pomace, with quercetin-3-galactoside, quercetin-3-glucoside, and quercetin-3-*O*-rutinoside as the most abundant, aligning with the present results, although they did not detect kaempferol-3-glucoside. Oszmiański and Lachowicz [4] also reported a lower flavonol content (1.5%) with the same major quercetin derivatives.

Phenolic acids accounted for 2.5% of the identified phenolics (2.41 mg/g dm), with neochlorogenic and chlorogenic acids nearly equally represented, while isochlorogenic acid A was slightly less abundant. These results are in line with those of Sójka et al. [5], who found that phenolic acids constituted 1.5% of chokeberry pomace phenolics, with similar concentrations of chlorogenic and neochlorogenic acids. Oszmiański and Lachowicz [4] reported a higher proportion of phenolic acids (7.5%) but also similar relative concentrations of these acids.

Flavan-3-ols and procyanidins were the least abundant, representing only 0.2% and 0.04% of total identified phenolics, respectively. Although present in low concentrations, flavan-3-ols and procyanidins are recognized as potent antioxidants that significantly contribute to the overall antioxidant capacity of plant materials [43]. Oszmiański and Lachowicz [4] found that flavan-3-ols accounted for 1.3% of TP in chokeberry pomace, with epicatechin at higher levels (2.601 mg/g dry matter) and procyanidin B2 at lower levels.

Overall, the composition of individual phenolic compounds in chokeberry pomace reflects the influence of cultivar, pomace preparation, extraction optimization, and analytical method, emphasizing the importance of standardized processing and precise analytical techniques for accurate phenolic profiling.

## 3. Materials and Methods

### 3.1. Chemicals and Reagents

Distilled water was obtained by a Milli-Q water purification system (Millipore, Bedford, MA, USA). Ethanol (96%), iron (III) chloride hexahydrate, potassium chloride, and methanol were procured from Gram-mol (Zagreb, Croatia). Hydrochloric acid (37%) and glacial acetic acid were purchased from Carlo Erba Reagents (Val-de-Reuil, France), while the Folin–Ciocolateu reagent was obtained from Merck (Darmstandt, Germany). Sodium carbonate (anhydrous), sodium acetate trihydrate, and potassium persulfate were from Kemika (Zagreb, Croatia), and acetonitrile and fluorescein from Honeywell Riedel-de-Haën (Seelze, Germany). 2,4,6-tri(2-pyridyl)-s-triazine (TPTZ), 6-hydroxy-2,5,7,8-tetramethylchroman-2-carboxylic acid (Trolox), 2,2-diphenyl-1-(2,4,6-trinitrophenyl)hydrazyl (DPPH), 2,2′-azino-bis(3-ethylbenzothiazoline-6-sulfonic acid (ABTS), and 2,2′-Azobis(2-amidinopropane) dihydrochloride (AAPH) were from Sigma-Aldrich (St. Louis, MO, USA) and formic acid was from BDH Prolabo (Lutterworth, UK). Working standards for gallic acid, cyanindin-3-glucoside chloride, chlorogenic acid, quercetin-3-glucoside, kaempferol-3-*O* glucoside, and catechin were procured from Sigma-Aldrich.

### 3.2. Plant Material

Frozen black chokeberries (*A. melanocarpa* L.), harvested in 2023, were procured from Terra Food Ltd. (Koprivnica, Croatia). Upon receipt, the berries were immediately thawed at room temperature and processed into juice by pressing in a juice extractor (Hurom HU-100, Ver Vita Ltd., Zagreb, Croatia). The remaining BCP was collected and frozen at −80 °C/24 h before being freeze-dried at − 55 °C/48 h (Alpha 1-4 LSCPlus, Martin Christ Gefriertrocknungsanlagen GmbH, Osterode am Harz, Germany). The freeze-dried BCP was then packaged into polypropylene bags, vacuumed and hermetically sealed, and stored in a desiccator at room temperature for further analysis. The total solid content of the freeze-dried BCP was 97.79%, as determined using a moisture analyzer (Ohaus MB25, Parsippany, NJ, USA). Immediately prior to extraction, freeze-dried BCP used for the determination of optimal PLE, MAE, and UAE process conditions and for reflux extraction was ground using conventional grinding in an electric grinding machine (Waring WSG30, Sprzęt Laboratoryjny i Medyczny Labpartner KBS, Warszawa, Poland), while the freeze-dried BCP used for the determination of the effect of cryogrinding was ground using cryogenic liquid.

### 3.3. Extraction Procedure

#### 3.3.1. PLE

The Dionex^TM^ASE^TM^ 350 Accelerated Solvent Extractor (Thermo Fisher Scientific Inc., Sunnyvale, CA, USA) was used for the PLE of polyphenols and anthocyanins from freeze-dried BCP. Extractions were performed according to an experimental design (Table 1) that included variation of temperature (100, 125, and 150 °C), static extraction time (5 and 10 min), and SSR (1:40, 1:60, and 1:80 g/mL) using a 1% formic acid in 50% aqueous ethanol solution as extraction solvent. The procedure was performed in stainless steel cells (34 mL) fitted with two cellulose filters at the bottom and one at the top, and filled with a mixture of the appropriate amount of sample and diatomaceous earth at constant parameters: 10.34 MPa pressure, 3 extraction cycles, 30 sec nitrogen purge, and 30% volume flush while varying the parameters temperature and static extraction time as described above. The extracts obtained were filtered into a volumetric flask (50 mL) and filled with the extraction solvent. Afterwards, they were stored in plastic test tubes at +4 °C until analysis.

#### 3.3.2. MAE

The MAE of BCP polyphenols and anthocyanins was performed with an Ethos Easy Reactor (Milestone, Sorisole, Italy). An appropriate amount of ground freeze-dried BCP was added to the extraction cell together with 20 mL of solvent (1% formic acid in 50% aqueous ethanol solution) and a magnetic stirrer. The sealed cell was then placed in the reactor, and the extractions were carried out under the experimental design data (Table 1), varying the temperature (40, 60, and 80 °C; constant throughout procedure), extraction time (5 and 10 min), and SSR (1:40, 1:60 and 1:80 g/mL), while the microwave power (400 W), preheating time (2 min for 40 °C, 4 min for 60 °C, and 6 min for 80 °C), post-extraction aeration and cooling (2 min), and stirring intensity (50%) were kept constant. The extracts obtained were filtered into volumetric flasks (25 mL), filled with a solvent, and stored in plastic test tubes at + 4 °C until analysis.

#### 3.3.3. UAE

The ultrasound processor UP200Ht (Dr. Hielscher GmbH, Teltow, Germany; max. output power 200 W, ultrasound frequency 26 kHz) was used for the UAE of polyphenols and anthocyanins from BCP. An appropriate amount of ground freeze-dried BCP and 20 mL of solvent (1% formic acid in 50% aqueous ethanol solution) were mixed in a glass beaker (100 mL) and placed in an ice bath to ensure a maximum temperature of 30 °C during the extraction. The extractions were performed by immersing a titanium ultrasound probe (154 mm^2^) 1 cm deep into the extraction mixture, following the experimental design (Table 1). The parameters varied were the ultrasound amplitude (50, 75, and 100%), the extraction time (5 and 10 min), and the SSR (1:40, 1:60, and 1:80 g/mL). During extraction, the temperature was monitored with an infrared thermometer. After extraction, the extracts were filtered into volumetric flasks (25 mL), filled with a solvent, and stored in plastic test tubes at + 4 °C until analysis.

#### 3.3.4. Reflux

Conventional heat extraction with reflux was carried out by weighing 0.5 g of ground freeze-dried BCP into a round-bottom flask and mixing it with 40 mL of extraction solvent (1% formic acid in 50% aqueous ethanol solution). The extraction mixture was then heated under reflux for 30 min from the start of boiling. The resulting extract was filtered into a volumetric flask (50 mL), made up with a solvent, and stored in a plastic test tube at +4 °C until analysis.

### 3.4. Cryogrinding

For pre-treatment by cryogrinding, 15 g of freeze-dried BCP was weighed into the grinding cell (50 mL), together with a 10 cm stainless steel pin. The cell was then sealed with a stainless cap and ground in a laboratory-scale Spex 6875D Freezer/Mill (Metuchen, NJ, USA) under a stream of liquid nitrogen (Messer, Zagreb, Croatia). Prior to grinding, the sample was pre-cooled for 2 min. Cryogrinding was performed for 1, 3, 5, 7, and 9 min at a rate of 14 cycles per second (cps). The resulting powders were collected and stored in plastic test tubes at −18 °C until extraction.

### 3.5. Particle Size Measurement

Particle size analysis of the conventionally ground and cryoground BCP samples was performed using laser diffraction on the Malvern Mastersizer 2000 (Malvern Instruments, Worcestershire, UK). Data acquisition and processing were carried out using Mastersizer 2000 software (ver. 5.61). The measurements were conducted with the Scirocco dry dispersion unit, with approximately 3 g of powder loaded into the sample chamber of the dispersion unit. The measurements were performed in duplicate at a pressure of 1.5 bar and a feed rate of 100%. Values for the 10th, 50th, and 90th percentiles (d(0.1), d(0.5), and d(0.9); µm) of the cumulative size distribution of the powders were recorded under the following parameters: a refractive index of 1.5, absorption of 0.1, and obscuration level of 3%. Span values, representing the homogeneity of the particle size distribution, were calculated according to the equation

Span = (d(0.9) − d(0.1))/d(0.5)(1)

### 3.6. Determination of Total Polyphenols

The TP content was determined by the spectrophotometric Folin–Ciocalteu method described by Elez Garofulić et al. [44]. For this purpose, an appropriately diluted extract (100 μL), the Folin–Ciocalteu reagent (200 μL), and distilled water (2 mL) were mixed, and 1 mL of sodium carbonate solution (20%, *w*/*v*) was added after 3 min. The reaction mixture was vortexed and then thermostated in a water bath (50 °C). After 25 min, the absorbance was measured at 765 nm against the blank containing 100 μL of extraction solvent instead of the extract and the same reagents. The results were expressed in mg GAE/g dm based on the gallic acid calibration curve.

### 3.7. Determination of Total Monomeric Anthocyanins

The spectrophotometric pH differential method [45] was used for the determination of TMA content. In brief, extract (1 mL) was mixed with 0.025 M potassium chloride buffer (pH 1.0) (4 mL) or with 0.4 M sodium acetate buffer (pH 4.5) (4 mL) and kept at room temperature. After 20 min, the absorbance was measured at 520 and 700 nm using distilled water as a blank. The TMA content was calculated as C3GE (mg/L) according to the equation

TMA (mg/L) = (A · MW · DF · 10^3^)/(ε · l) (2)

where

A = (A_520nm_ − A_700nm_)_pH=1.0_ − (A_520nm_ − A_700nm_)_pH=4.5_;
MW = molecular weight of cyanidin-3-glucoside (449.2 g/mol);
DF = dilution factor;
10^3^ = factor for conversion g to mg;
ε = molar absorption extinction coefficient of cyanidin-3-glucoside (26,900 L/mol cm);
l = cuvette thickness (1 cm).

The final results were expressed in mg of C3GE/g dm.

### 3.8. Phenolic Characterization by UPLC-MS/MS

The individual polyphenols in the BCP extract obtained under optimal MAE conditions combined with cryogrinding pre-treatment were determined by UPLC-MS/MS analysis. Prior to analysis, the extract was filtered through a 0.45 µm membrane filter. The analysis took place on an Agilent 1290 series RRLC instrument with a 6430 triple quadrupole mass spectrometer (Agilent, Santa Clara, CA, USA) and an ESI ion source, according to the method described by Elez Garofulić et al. [46]. ESI ionization was carried out in both positive and negative modes (*m*/*z* 100 to 1000) using nitrogen (99.999%, Messer, Zagreb, Croatia) as an induction cone and collision gas at a positive/negative capillary voltage of 4000/3500 V, a drying gas temperature of 300 °C, a flow rate of 11 L/h, and nebulizer pressure of 40 psi. A Zorbax Eclipse Plus C18 column (Agilent, 100 × 2.1 mm; 1.8 µm particle size) at 35 °C was used to separate the compounds, with an injection volume of 2.5 µL, a flow rate of 0.3 mL/min, and the following eluents: eluent A—0.1% formic acid (*v*/*v*) in water and eluent B—0.1% formic acid in acetonitrile (*v*/*v*). Agilent MassHunter workstation software (ver. B.04.01) was used for data acquisition and processing. All other method quality parameters are described in detail in the authors’ study [46]. Identification and quantification were performed using calibration curves for the following standards: cyanindin-3-glucoside chloride, chlorogenic acid, quercetin-3-glucoside, kaempferol-3-*O*-glucoside, and catechin. For compounds lacking commercial reference standards, tentative identification was based on mass spectral data and literature reports of mass fragmentation patterns. Quantification involved using cyanindin-3-glucoside chloride calibration for cyanidin-3-*O*-galactoside, cyanidin-3-*O*-glucoside, cyanidin-3-*O*-arabinoside, and cyanidin-3-*O*-xyloside, chlorogenic acid calibration for neochlorogenic acid and isochlorogenic acid A, quercetin-3-glucoside calibration for quercetin-3-*O*-dihexoside, isorhamnetin-3-rutinoside, quercetin-3-*O*-rutinoside, isorhamnetin-pentosylhexoside, quercetin-3-*O*-vicianoside, isorhamnetin-3-*O*-glucoside, quercetin-3-*O*-glucuronide, isorhamnetin-3-*O*-galactoside, quercetin-3-*O*-galactoside and quercetin-3-glucoside, kaempferol-3-*O* glucoside calibration for kaempferol-3-rutinoside, and catechin calibration for epicatechin and procyanidin B2. All results were expressed in mg/g dm.

### 3.9. Determination of Antioxidant Capacity

#### 3.9.1. Ferric Reducing Antioxidant Power (FRAP) Analysis

The FRAP analysis method by Benzie and Strain [47], with some modifications, was followed. Briefly, a mixture of distilled water (240 μL), appropriately diluted extract (80 μL), and FRAP reagent (a mixture of sodium acetate buffer (0.3 M, pH 3.6), 10 mM TPTZ solution in 40 mM hydrochloric acid, and 20 mM FeCl_3_·6H_2_O aqueous solution in the ratio 10:1:1) (2080 μL) was vortexed and thermostatted at 37 °C. After 5 min, the absorbance was measured at 593 nm against the blank containing 80 μL of extraction solvent instead of the extract and with the same reagents. The results were expressed as µmol of Trolox equivalents (TE)/g dm based on the Trolox calibration curve.

#### 3.9.2. 2,2-Diphenyl-1-picrylhydrazyl Radical (DPPH) Scavenging Analysis

DPPH method was performed using the method of Braca et al. [48]: 0.75 mL of the appropriately diluted extract was combined with 1.5 mL of 0.2 mM DPPH solution and left in the dark at room temperature for 20 min. Afterwards, the absorbance was measured at 517 nm against the blank (100% methanol). The results were expressed as µmol TE/g dm based on the Trolox calibration curve.

#### 3.9.3. 2,2-Azinobis(3-ethylbenzothiazoline-6-sulfonic Acid) (ABTS) Analysis

The ABTS antioxidant capacity was determined according to the modified method of Miller and Rice-Evans [49]. For this purpose, an aliquot of 160 μL of appropriately diluted extract and 2 mL of 1% ABTS^•+^ solution were mixed and left for 1 min to incubate. Then, the absorbance was measured at 734 nm against the blank (96% ethanol). The results were expressed as μmol TE/g dm according to the Trolox calibration curve.

#### 3.9.4. Oxygen Radical Absorbance Capacity (ORAC) Analysis

The ORAC analysis method described by Elez Garofulić et al. [50] was applied using an automated 96-well microplate fluorescence plate reader (Clariostar, BMG LABTECH, Offenburg, Germany). The appropriately diluted extract was placed in a black plate containing a fluorescein solution (70.3 nM) and incubated at 37 °C for 30 min. After the first three cycles (representing the baseline signal), 240 mM AAPH was injected into each well to generate the peroxyl radical. Different Trolox dilutions (3.12–103.99 μM) were used in each plate as a reference standard, and fluorescence intensity (excitation at 485 nm and emission at 528 nm) was monitored every 90 s over a total measurement period of 120 min. Data were analyzed by MARS 2.0 software, and the results were expressed as μmol TE/g dm according to the Trolox calibration curve.

### 3.10. Experimental Design and Statistical Analysis

The Statistica 12.0 software system (StatSoft, Inc., Tulsa, OK, USA) was used for the experimental design and statistical data processing. In the first part of the study, i.e., optimizing the conditions for PLE, MAE, and UAE, all experiments were designed as mixed full-factorial experimental designs, with three independent factors at two (extraction time) or three levels (extraction temperature or amplitude and SSR) for each extraction technique, while the dependent variables were the content of TP (mg GAE/g dm) and TMA (mg C3GE/g dm). The normality and homogeneity of the distribution of residual data were tested with the Shapiro–Wilks and Levene tests, where normally distributed and homogeneous data were analyzed with a parametric test of multifactorial analysis of variance (ANOVA), and the comparison of marginal means was performed with the post hoc Tukey test. Data that deviated from the normal distribution and were inhomogeneous were analyzed using the non-parametric Kruskal–Wallis test. The results of this statistical analysis are expressed as mean ± SE. Furthermore, to examine the possible separation of extracts according to extraction technique, a PCA was applied to the correlation matrix comprising 54 samples and data for the content of TP and TMA (108 data points in total), considering principal components with an eigenvalue > 1 and including variables with a communality value ≥ 0.5 in the analysis.

In the second part of the study, i.e., when comparing the efficiency of PLE, MAE, UAE and reflux extraction and the effect of cryogrinding time, the experiments were designed as a single-factor design in which one independent factor at four (PLE, MAE, UAE, and reflux) or five levels (1, 3, 5, 7, and 9 min) was observed with the same dependent variables as in the first part of the study. The influence of extraction technique or duration of cryogrinding on the efficiency of extraction of polyphenols and anthocyanins and antioxidant capacity (FRAP, DPPH, ABTS, ORAC; µmol TE/g dm) was analyzed using the same statistical methodology as described above. The results of this statistical analysis are expressed as mean ± SD. All statistical tests were based on the significance level *p* ≤ 0.05.

## 4. Conclusions

In this study, the efficiency of PLE, MAE, and UAE for recovering TP and TMA from BCP was systematically evaluated and compared to conventional reflux extraction. The results clearly demonstrate that process parameters, particularly temperature, time, and SSR, play critical roles in maximizing bioactive compound yield and antioxidant capacity. Among the advanced techniques, PLE proved the most effective for maximizing TP content, benefiting from higher temperatures that enhance the extraction process, though it showed limitations in preserving thermolabile anthocyanins. In contrast, MAE emerged as the most balanced method overall, combining relatively high yields of both TP and TMA with notable antioxidant activity across multiple assays, likely due to efficient cell disruption at moderate temperatures and short processing times. UAE showed lower efficiency in TP extraction under the tested conditions, but maintained competitive TMA yields, especially considering its mild operational parameters. The integration of cryo-grinding as a pre-treatment notably enhanced TP recovery by reducing particle size and increasing surface area for mass transfer, with just 1 min of cryogrinding achieving abundant improvements. The phenolic profile analysis of optimized extracts confirmed that anthocyanins, particularly cyanidin-3-glucoside and cyanidin-3-galactoside, dominate the BCP matrix, complemented by flavonols, phenolic acids, and minor flavan-3-ols and procyanidins, altogether contributing to the strong antioxidant potential observed. When benchmarked against reflux extraction, advanced methods, especially MAE combined with cryogrinding, offer comparable or superior extraction efficiency while providing significant advantages in reduced processing time that aligns well with the principles of sustainable green extraction technologies. Importantly, this research highlights the substantial potential of BCP, a major by-product of juice production and typically underutilized, as a valuable source of health-promoting compounds. By optimizing modern green extraction technologies and integrating simple yet effective pre-treatments like cryogrinding, this study demonstrates a viable approach to valorizing agro-industrial residues, reducing food processing waste, and contributing to sustainable bio-economy models. Such strategies align with global efforts to promote resource efficiency, support circular economy principles, and develop value-added functional ingredients for the food and nutraceutical sectors.

## Figures and Tables

**Table 1:** Content of TP and TMA in BCP extracts obtained under PLE, MAE, and UAE.

Extraction Technique	Temperature (°C)	Amplitude (%)	Time (min)	SSR (g/mL)	TP (mg GAE/g dm)	TMA (mg C3GE/g dm)
PLE	100		5	1:40	86.0 ± 0.2	22.5 ± 0.1
	100		5	1:60	101.6 ± 1.6	24.1 ± 0.1
	100		5	1:80	113.1 ± 4.6	23.9 ± 0.3
	100		10	1:40	99.2 ± 4.6	22.4 ± 0.1
	100		10	1:60	108.6 ± 5.6	21.5 ± 0.5
	100		10	1:80	118.1 ± 6.7	20.6 ± 0.1
	125		5	1:40	101.3 ± 8.7	19.3 ± 0.2
	125		5	1:60	97.6 ± 3.0	18.2 ± 0.3
	125		5	1:80	115.6 ± 0.8	18.7 ± 0.1
	125		10	1:40	110.4 ± 4.4	15.0 ± 0.1
	125		10	1:60	119.2 ± 3.4	15.6 ± 0.4
	125		10	1:80	108.7 ± 1.0	16.2 ± 0.1
	150		5	1:40	119.8 ± 1.3	13.7 ± 0.3
	150		5	1:60	123.1 ± 5.0	14.1 ± 0.1
	150		5	1:80	128.7 ± 0.2	13.4 ± 0.1
	150		10	1:40	126.2 ± 4.2	12.0 ± 0.1
	150		10	1:60	123.9 ± 3.9	12.3 ± 0.2
	150		10	1:80	132.5 ± 7.4	8.2 ± 0.1
MAE	40		5	1:40	58.5 ± 1.5	20.7 ± 0.4
	40		5	1:60	69.3 ± 5.9	23.7 ± 0.2
	40		5	1:80	75.5 ± 1.0	25.9 ± 0.7
	40		10	1:40	63.5 ± 0.4	23.1 ± 1.3
	40		10	1:60	72.7 ± 2.1	24.3 ± 0.8
	40		10	1:80	79.8 ± 1.8	26.6 ± 0.1
	60		5	1:40	65.4 ± 1.7	20.8± 0.1
	60		5	1:60	72.8 ± 1.8	24.5 ± 0.5
	60		5	1:80	81.3 ± 4.5	24.5 ± 0.1
	60		10	1:40	70.6 ± 0.1	22.7 ± 0.2
	60		10	1:60	75.5 ± 2.7	23.8 ± 0.5
	60		10	1:80	90.1 ± 4.3	25.6 ± 0.2
	80		5	1:40	68.9 ± 0.8	20.3 ± 0.2
	80		5	1:60	84.7 ± 2.0	22.3 ± 0.5
	80		5	1:80	82.4 ± 6.7	24.8 ± 0.1
	80		10	1:40	76.1 ± 1.2	22.7 ± 0.2
	80		10	1:60	95.7± 6.52	25.2 ± 0.4
	80		10	1:80	94.8 ± 8.3	24.0 ± 0.5
UAE		50	5	1:40	65.2 ± 2.2	20.9 ± 0.7
		50	5	1:60	79.9 ± 2.7	23.2 ± 0.9
		50	5	1:80	75.2 ± 1.5	22.4 ± 0.4
		50	10	1:40	69.7 ± 1.0	20.8 ± 0.1
		50	10	1:60	74.4 ± 4.3	20.4 ± 0.1
		50	10	1:80	73.9 ± 1.8	20.5 ± 0.5
		75	5	1:40	70.6 ±2.8	21.7 ± 0.2
		75	5	1:60	75.3 ± 6.6	21.0 ± 0.1
		75	5	1:80	74.3 ± 1.2	21.5 ± 0.3
		75	10	1:40	66.7 ± 1.0	21.0 ± 0.1
		75	10	1:60	71.7 ± 1.6	22.6 ± 0.3
		75	10	1:80	84.1 ± 2.2	21. 9 ± 0.1
		100	5	1:40	64.8 ± 2.2	20.2 ± 0.1
		100	5	1:60	75.7 ± 3.6	22.5 ± 0.4
		100	5	1:80	74.6 ± 5.7	21.3 ± 0.1
		100	10	1:40	63.1 ± 0.4	19.9 ± 1.3
		100	10	1:60	69.8 ± 5.7	18.7 ± 0.2
		100	10	1:80	88.9 ± 2.0	22.2 ± 0.1

TP—total polyphenols; TMA—total monomeric anthocyanins; BCP—black chokeberry pomace; PLE—pressurized liquid extraction; MAE—microwave-assisted extraction; SSR—solid–solvent ratio; GAE—gallic acid equivalents; C3GE—cyanidin-3-glucoside equivalents. Results are expressed as mean ± standard deviation (SD).

**Table 2:** The influence of PLE, MAE, and UAE process conditions on TP and TMA in BCP extracts.

Source of Variation	TP (mg GAE/g dm)	TMA (mg C3GE/g dm)
PLE		
Temperature (°C)	*p* < 0.001 *	*p* < 0.001 *
100	104.4 ± 3.3 ^a^	22.5 ± 0.4 ^c^
125	108.8 ± 2.5 ^a^	17.3 ± 0.5 ^b^
150	125.7 ± 1.5 ^b^	12.3 ± 0.6 ^a^
Time (min)	*p* = 0.155	*p* = 0.070
5	109.6 ± 3.2 ^a^	18.7 ± 1.0 ^a^
10	116.3 ± 2.5 ^a^	16.0 ± 1.1 ^a^
SSR (g/mL)	*p* = 0.053	*p* = 0.953
1:40	107.2 ± 4.2 ^a^	17.5 ± 1.3 ^a^
1:60	112.3 ± 3.2 ^a^	17.7 ± 1.3 ^a^
1:80	119.4 ± 2.7 ^a^	16.8 ± 1.5 ^a^
MAE		
Temperature (°C)	*p* = 0.003 *	*p* = 0.552
40	69.9 ± 2.2 ^a^	24.0 ± 0.6 ^a^
60	76.0 ± 2.5 ^ab^	23.6 ± 0.5 ^a^
80	83.8 ± 3.1 ^b^	23.2 ± 0.5 ^a^
Time (min)	*p* = 0.050 *	*p* = 0.054
5	73.2 ± 2.1 ^a^	23.1 ± 0.5 ^a^
10	79.9 ± 2.7 ^b^	24.2 ± 0.3 ^a^
SSR (g/mL)	*p* < 0.001 *	*p* < 0.001 *
1:40	67.2 ± 1.7 ^a^	21.7 ± 0.4 ^a^
1:60	78.5 ± 2.9 ^b^	24.0 ± 0.3 ^b^
1:80	84.0 ± 2.2 ^b^	25.2 ± 0.3 ^c^
UAE		
Amplitude (%)	*p* = 0.944	*p* = 0.228
50	73.1 ± 1.5 ^a^	21.4 ± 0.3 ^a^
75	73.8 ± 1.8 ^a^	21.6 ± 0.2 ^a^
100	72.8 ± 2.7 ^a^	20.8 ± 0.4 ^a^
Time (min)	*p* = 0.749	*p* = 0.050 *
5	72.9 ± 1.3 ^a^	21.6 ± 0.2 ^b^
10	73.6 ± 1.9 ^a^	20.9 ± 0.3 ^a^
SSR (g/mL)	*p* < 0.001 *	*p* = 0.161
1:40	66.7 ± 0.9 ^a^	20.7 ± 0.2 ^a^
1:60	74.5 ± 1.4 ^b^	21.4 ± 0.5 ^a^
1:80	78.5 ± 1.9 ^b^	21.6 ± 0.2 ^a^

PLE—pressurized liquid extraction; MAE—microwave-assisted extraction; UAE—ultrasound-assisted extraction; TP—total polyphenols; TMA—total monomeric anthocyanins; BCP—black chokeberry pomace; SSR—solid–solvent ratio; GAE—gallic acid equivalents; C3GE—cyanidin-3-glucoside equivalents. Results are expressed as mean ± standard error (SE). * *p* ≤ 0.05. Means with the same letter within a column are not significantly different at *p* ≤ 0.05.

**Table 3:** Content of TP, TMA, and antioxidant capacity in BCP extracts obtained under PLE, MAE, and UAE optimal conditions and reflux extraction.

Extraction Technique	TP (mg GAE/g dm)	TMA (mg C3GE/g dm)	FRAP (µmol TE/g dm)	DPPH (µmol TE/g dm)	ABTS (µmol TE/g dm)	ORAC (µmol TE/g dm)
	*p* = 0.012 *	*p* = 0.007 *	*p* = 0.002 *	*p* = 0.001 *	*p* < 0.001 *	*p* < 0.001 *
PLE	101.3 ± 8.7 ^ab^	19.3 ± 0.2 ^a^	1052.1 ± 48.9 ^b^	297.1 ± 2.5 ^a^	519.2 ± 9.4 ^a^	92.2 ± 2.2 ^a^
MAE	94.8 ± 8.3 ^a^	24.0 ± 0.5 ^b^	983.7 ± 60.5 ^b^	420.4 ± 8.0 ^b^	681.3 ± 24.8 ^b^	199.6 ± 1.6 ^c^
UAE	79.9 ± 2.7 ^a^	23.2 ± 0.9 ^b^	637.4 ± 28.1 ^a^	315.7 ± 3.2 ^a^	461.3 ± 15.0 ^a^	142.7 ± 1.9 ^b^
REFLUX	120.8 ± 0.8 ^b^	22.9 ± 0.8 ^b^	1033.4 ± 16.7 ^b^	472.9 ± 25.3 ^b^	790.2 ± 14.4 ^c^	222.6 ± 2.9 ^d^

TP—total polyphenols; TMA—total monomeric anthocyanins; BCP—black chokeberry pomace; PLE—pressurized liquid extraction; MAE—microwave-assisted extraction; UAE—ultrasound-assisted extraction; GAE—gallic acid equivalents; C3GE—cyanidin-3-glucoside equivalents; TE—Trolox equivalents. Results are expressed as mean ± SD. * *p* ≤ 0.05. Means with the same letter within a column are not significantly different at *p* ≤ 0.05.

**Table 4:** Particle size of BCP powder obtained by conventional grinding and cryogrinding.

Cryogrinding Time (min)	d(0.1) (µm)	d(0.5) (µm)	d(0.9) (µm)	Span
	*p* < 0.001 *	*p* < 0.001 *	*p* < 0.001 *	*p* < 0.001 *
0	37.0 ± 2.8 ^b^	238.1 ± 10.9 ^d^	712.9 ± 43.4 ^c^	2.8 ± 0.0 ^a^
1	5.0 ± 0.2 ^a^	75.3 ± 2.3 ^c^	455.6 ± 17.3 ^b^	6.0 ± 0.1 ^b^
3	3.7 ± 0.1 ^a^	53.1 ± 4.2 ^b^	419.7 ± 70.1 ^b^	7.8 ± 0.7 ^bc^
5	2.6 ± 0.1 ^a^	24.9 ± 1.1 ^a^	245.1 ± 29.2 ^a^	9.7 ± 0.7 ^c^
7	2.3 ± 0.1 ^a^	19.4 ± 0.7 ^a^	186.7 ± 19.8 ^a^	9.5 ± 0.7 ^c^
9	2.1 ± 0.1 ^a^	15.4 ± 0.8 ^a^	135.6 ± 13.5 ^a^	8.7 ± 0.5 ^c^

BCP—black chokeberry pomace. Results are expressed as mean ± SD. * *p* ≤ 0.05. Means with the same letter within a column are not significantly different at *p* ≤ 0.05.

**Table 5:** Content of TP and TMA and antioxidant capacity in BCP extracts obtained under MAE optimal conditions in combination with cryogrinding pre-treatment.

Cryogrinding Time (min)	TP (mg GAE/g dm)	TMA (mg C3GE/g dm)	FRAP (µmol TE/g dm)	DPPH (µmol TE/g dm)	ABTS (µmol TE/g dm)	ORAC (µmol TE/g dm)
	*p* = 0.001 *	*p* = 0.980	*p* = 0.003 *	*p* = 0.046 *	*p* = 0.004 *	*p* = 0.006 *
1	115.1 ± 0.2 ^b^	22.0 ± 0.7 ^a^	816.5 ± 6.7 ^ab^	380.3 ± 3.3 ^a^	631.5 ± 11.7 ^a^	204.1 ± 4.1 ^b^
3	103.4 ± 0.7 ^a^	22.3 ± 0.9 ^a^	822.8 ± 2.2 ^ab^	395.3 ± 4.7 ^ab^	634.6 ± 7.3 ^a^	192.6 ± 3.4 ^b^
5	100.7 ± 2.5 ^a^	22.2 ± 0.9 ^a^	945.2 ± 13.5 ^c^	403.9 ± 4.4 ^ab^	651.3 ± 16.0 ^ab^	190.9 ± 1.1 ^ab^
7	99.1 ± 0.2 ^a^	22.4 ± 0.7 ^a^	786.6 ± 22.4 ^a^	401.3 ± 13.1 ^ab^	688.4 ± 13.1 ^bc^	189.6 ± 4.4 ^ab^
9	104.2 ± 2.5 ^a^	22.1 ± 0.4 ^a^	881.5 ± 35.9 ^bc^	411.7 ± 5.5 ^b^	718.1 ± 14.6 ^c^	177.1 ± 4.0 ^a^

TP—total polyphenols; TMA—total monomeric anthocyanins; BCP—black chokeberry pomace; MAE—microwave-assisted extraction; GAE—gallic acid equivalents; C3GE—cyanidin-3-glucoside equivalents; TE—Trolox equivalents. Results are expressed as mean ± SD. * *p* ≤ 0.05. Means with the same letter within a column are not significantly different at *p* ≤ 0.05.

**Table 6:** Polyphenol composition in BCP extract obtained under optimal MAE conditions in combination with 1 min of cryogrinding pre-treatment.

Polyphenol	Precursor Ion (*m*/*z*)	Product Ion (*m*/*z*)	mg/g dm
Anthocyanins			
Cyanidin-3-*O*-galactoside	449	287	20.67 ± 0.03
Cyanidin-3-*O*-glucoside	449	287	20.81 ± 0.01
Cyanidin-3-*O*-arabinoside	419	287	11.37 ± 0.06
Cyanidin-3-*O*-xyloside	419	287	11.31 ± 0.04
		Total	64.16
Phenolic acids			
Isochlorogenic acid A	515	353	0.70 ± 0.01
Chlorogenic acid	353	191	0.80 ± 0.04
Neochlorogenic acid	353	191	0.91 ± 0.02
		Total	2.41
Flavonols			
Quercetin-3-*O*-dihexoside	627	303	0.15 ± 0.01
Isorhamnetin-3-rutinoside	625	317	0.06 ± 0.01
Quercetin-3-*O*-rutinoside	611	303	0.91 ± 0.02
Isorhamnetin-pentosylhexoside	611	317	0.02 ± 0.04
Quercetin-3-*O*-vicianoside	597	434, 303	0.16 ± 0.01
Kaempferol-3-rutinoside	595	287	0.03 ± 0.05
Isorhamnetin-3-*O*-glucoside	479	317	0.02 ± 0.01
Quercetin-3-*O*-glucuronide	479	303	0.03 ± 0.02
Isorhamnetin-3-*O*-galactoside	479	317	0.02 ± 0.02
Quercetin-3-*O*-galactoside	465	303	1.54 ± 0.04
Quercetin-3-glucoside	465	303	1.61 ± 0.03
Kaempferol-3-glucoside	449	287	24.53 ± 0.03
		Total	29.08
Flavan-3-ols and Procyanidins			
Epicatechin	291	139, 123	0.22 ± 0.05
Procyanidin B2	577	289	0.04 ± 0.04
		Total	0.26
Total UPLC-MS/MS polyphenols			95.91

BCP—black chokeberry pomace; MAE—microwave-assisted extraction. Results are expressed as mean ± SD.

## Data Availability

The original contributions presented in the study are included in the article material; further inquiries can be directed to the corresponding author.

## Abbreviations

The following abbreviations are used in this manuscript:

BCP	Black chokeberry pomace
PLE	Pressurized liquid extraction
MAE	Microwave-assisted extraction
UAE	Ultrasound-assisted extraction
TP	Total polyphenols
TMA	Total monomeric anthocyanins
SSR	Solid–solvent ratio
PCA	Principal component analysis
PC1, PC2	Principal components
